# Hepcidin Upregulation in Lung Cancer: A Potential Therapeutic Target Associated With Immune Infiltration

**DOI:** 10.3389/fimmu.2021.612144

**Published:** 2021-04-01

**Authors:** Yumei Fan, Bing Liu, Fei Chen, Zhiyuan Song, Bihui Han, Yanxiu Meng, Jiajie Hou, Pengxiu Cao, Yanzhong Chang, Ke Tan

**Affiliations:** ^1^ Key Laboratory of Animal Physiology, Biochemistry and Molecular Biology of Hebei Province, College of Life Sciences, Hebei Normal University, Shijiazhuang, China; ^2^ Department of Neurosurgery, HanDan Central Hospital, Handan, China

**Keywords:** hepcidin, lung cancer, prognostic biomarker, immune infiltration, iron

## Abstract

Lung cancer has the highest death rate among cancers globally. Hepcidin is a fascinating regulator of iron metabolism; however, the prognostic value of hepcidin and its correlation with immune cell infiltration in lung cancer remain unclear. Here, we comprehensively clarified the prognostic value and potential function of hepcidin in lung cancer. Hepcidin expression was significantly increased in lung cancer. High hepcidin expression was associated with sex, age, metastasis, and pathological stage and significantly predicted an unfavorable prognosis in lung cancer patients. Gene Ontology (GO), Kyoto Encyclopedia of Genes and Genomes (KEGG) and Gene Set Enrichment Analysis (GSEA) results suggested that hepcidin is involved in the immune response. Furthermore, hepcidin expression was positively correlated with the infiltration levels of immune cells and the expression of diverse immune cell marker sets. Importantly, hepcidin may affect prognosis partially by regulating immune infiltration in lung cancer patients. Hepcidin may serve as a candidate prognostic biomarker for determining prognosis associated with immune infiltration in lung cancer.

## Introduction

Lung cancer is the leading cause of cancer-related death worldwide. Approximately 2.1 million new cases of lung cancer were diagnosed, and 1.8 million deaths were predicted in 2018 according to Global Cancer Statistics (1). Based on histological features, non-small-cell lung cancer (NSCLC) accounts for 80-85% of lung cancers and mainly includes lung squamous cell carcinoma (LUSC), lung adenocarcinoma (LUAD), and large-cell carcinoma (LCC) (2). Most NSCLC patients are diagnosed at late stages due to the absence of early typical clinical symptoms and effective diagnostic methods (3). Despite improvements in surgery and targeted therapeutic drugs, these current treatments still fail to yield desirable survival in lung cancer patients (2, 3). Therefore, there is a pressing need to explore novel prognostic predictors and therapeutic targets for lung cancer (4).

Iron is the most abundant trace element and plays critical roles in multiple cellular functions (5). In recent years, iron metabolism has attracted great attention as a mechanism in tumorigenesis (6–8). Among the regulators of iron homeostasis, hepcidin is thought to play an important role (9, 10). Hepcidin is a small (25-amino acid) antimicrobial regulator that prevents iron absorption by enterocytes, iron release from macrophages, and iron transport across the placenta (9, 10). The role of hepcidin is shown to be related to its regulation of the iron transporter ferroportin (FPN1). FPN1 is an important mediator of iron metabolism and is the only known iron exporter in mammals that transfers intracellular iron to the extracellular environment (5). Hepcidin can bind to FPN1 on the cell surface and cause internalization and ubiquitin-dependent degradation of FPN1, which increases intracellular iron levels (11, 12). When hepcidin expression is chronically increased, persistent hypoferremia can result in the development of iron-restricted anemia (13). In contrast, chronic hepcidin deficiency leads to excessive iron absorption, increased levels of nontransferrin-bound iron in circulation, and the development of hyperferremia-related diseases, such as hemochromatosis (13). Consistently, transgenic mice overexpressing hepcidin exhibit iron-deficient anemia, whereas hepcidin-deficient mice show iron overload in many organs (14, 15). Because of its critical role in mediating iron homeostasis and the pathogenesis of iron disorders, hepcidin has emerged as a promising drug target.

Hepcidin is a pivotal peptide hormone that exhibits bactericidal and fungicidal properties *in vitro* (16). It is prominently produced in the liver, released into plasma and excreted in urine (17). The expression of hepcidin is mainly regulated by iron excess, hypoxia, and inflammatory stimuli (16–18). Hepcidin synthesis is significantly induced by infection and inflammation. The upregulation of hepcidin by inflammation is regulated, at least in part, by the inflammatory cytokine interleukin-6 (IL-6), a major mediator of the acute phase response in hepatocytes (19, 20). IL-6 treatment promoted the expression of hepcidin in isolated hepatocytes and hepatoma cell lines through the Jak/signal transducer and activator of transcription (STAT) pathway (19, 20). Hepcidin expression did not increase in mice lacking IL-6 when treated with LPS. Administration of IL-6 to mice and human volunteers increased hepcidin production and led to hypoferremia (21). Importantly, bone morphogenic protein (BMP)/sma and mothers against the decapentaplegic homologue (SMAD) pathway contribute to the maximal induction of hepcidin by inflammation (19, 20). Moreover, IL-1 also upregulated hepcidin mRNA expression in mouse primary hepatocytes from both wild-type and IL-6 knockout mice, indicating that IL-1 may play an IL-6-independent role in the upregulation of hepcidin by inflammation (22). Therefore, cross talk may exist between different pathways regulating hepcidin expression. Because inflammation is often accompanied by tumorigenesis and hepcidin is closely related to inflammation, the relationship between hepcidin, inflammation and tumors remains to be further explored.

A growing body of studies has revealed that dysregulation of iron homeostasis is one of the metabolic hallmarks of cancer cells, illustrating that iron is required for tumor development, progression and metastasis (6–8). Consistent with this notion, previous studies have demonstrated that hepcidin expression is upregulated in several types of cancer, including breast cancer, renal cell carcinoma (RCC), pancreatic cancer, prostate cancer, and colorectal cancer (23). In breast cancer patients, hepcidin expression in both serum and cancer tissues is significantly upregulated compared with that in normal individuals (24, 25). Hepcidin exhibits diagnostic value in both breast cancer and breast cancer with bone metastasis (26). Furthermore, increased levels of hepcidin are also involved in the development of the malignant phenotype of breast cancer cells and resistance to doxorubicin (27). Hepcidin mRNA expression is higher in RCC patients with metastasis than in those without metastasis (28). High hepcidin expression is significantly correlated with poor survival in RCC patients (29). In pancreatic cancer, high expression of hepcidin is significantly associated with a poor prognosis in patients (30). Moreover, hepcidin expression is also associated with the pathological stage and vascular invasion of pancreatic cancer (30). The synthesis and secretion of hepcidin are also markedly increased in prostate cancer cells and tissues (31, 32). In addition, hepcidin expression is increased in colorectal cancer tissues compared to matched normal tissues and is related to advanced T stage (T3 and T4) (33). Therefore, hepcidin can serve as an independent risk factor and prognostic biomarker of different types of cancer (34–38). However, the role of hepcidin in lung cancer metastasis and its association with immune cell infiltration in lung cancer are less well understood.

Given the close relationship between iron homeostasis and tumorigenesis, limited evidence has illustrated the function and clinical significance of hepcidin in lung cancer pathogenesis and prognosis. The present study aims to integrate multiple bioinformatics approaches to investigate whether hepcidin is involved in lung cancer metastasis and immune infiltration and to explore its molecular regulation. We found that hepcidin expression was significantly upregulated in lung cancer tissues compared with nontumor tissues. Moreover, hepcidin expression was increased in tumor stages and correlated with axillary lymph node metastasis. High expression of hepcidin was negatively correlated with the prognosis of lung cancer patients. In addition, there was a significant relationship between the expression of hepcidin and the infiltration levels of B cells, CD4+ T cells, CD8+ T cells, macrophages, neutrophils, and dendritic cells in lung cancer. Importantly, hepcidin seemed to affect the prognosis of lung cancer patients partially through immune cell infiltration. These observations emphasize a noticeable role of hepcidin in carcinogenesis and indicate that hepcidin may play an important role in the regulation of immune cell infiltration in lung cancer.

## Materials and Methods

### Oncomine

Oncomine (www.oncomine.org) is a gene chip-based database for facilitating data mining of the transcriptional expression of genes in various cancers. The mRNA level of hepcidin in lung cancer was examined using Oncomine. The P-value was set as 0.05, the fold-change was set as 1.5, and the gene rank was set as all.

### UALCAN

UALCAN (http://ualcan.path.uab.edu/) is a web-based tool that provides in-depth analyses of transcriptome data from The Cancer Genome Atlas (TCGA) and MET500 data. UALCAN was used to investigate hepcidin expression and the association between hepcidin and various clinicopathological parameters (sex, cancer stages, nodal metastasis status, age, race and TP53 mutation status) of lung cancer.

### Gene Expression Profiling Interactive Analysis (GEPIA)

GEPIA (http://gepia.cancer-pku.cn/index.html) is a user-friendly web portal for gene expression analysis based on TCGA and GTEx data. In the current study, expression analysis of hepcidin was evaluated using TCGA-LUAD and TCGA-LUSC datasets. In the module “Expression DIY” of GEPIA, the expression of hepcidin between LUAD/LUSC and normal adjacent lung tissue samples was investigated with the option of matching TCGA normal and GTEx data and log2(TPM+1) for log-scale. Additionally, the relationships between hepcidin and PD-1, PD-L1 and CTLA-4 were determined using Spearman’s correlation coefficient in “correlation analysis”.

### cBioPortal

The cBioPortal for Cancer Genomics contains a large-scale cancer genomics dataset and has functions such as visualization, download, and analysis. We chose three lung cancer datasets with 2197 cases for further analysis by using cBioPortal. The genomic alteration types and alteration frequency of hepcidin in lung cancer were analyzed through the “OncoPrint” module and “Cancer Types Summary” module. The OS and disease-free survival (DFS) of hepcidin were analyzed through the “Comparison/Survival” module in cBioPortal.

### Gene Ontology (GO) Term and Kyoto Encyclopedia of Genes and Genomes (KEGG) Pathway Enrichment Analysis and Gene Set Enrichment Analysis (GSEA)

GO and KEGG analyses were applied to explore the biological functions of hepcidin in lung cancer. GO analysis is a powerful bioinformatics tool to determine the biological processes (BPs), cellular components (CCs) and molecular functions (MFs) related to hepcidin. GSEA was used to investigate the potential mechanisms of hepcidin. GO, KEGG and GSEA were performed by the R package ClusterProfiler.

### Tumor Immune Estimation Resource (TIMER)

TIMER (https://cistrome.shinyapps.io/timer/), an interactive web portal, could perform comprehensive analysis on the infiltration levels of different immune cells. In the present study, hepcidin expression in multiple types of cancer was evaluated through the “Diff Exp” module. The correlation of hepcidin and immune cell infiltration in LUAD and LUSC was analyzed in TIMER. The “Gene” module can investigate the relationship between hepcidin expression and immune cell infiltration levels (B cells, CD8+ T cells, CD4+ T cells, neutrophils, macrophages, and dendritic cells) using the TCGA database. TIMER was also applied to investigate the relationship between hepcidin expression and different gene marker sets of immune cells by using the “Correlation” module. The correlations of hepcidin expression with immune infiltration were evaluated by purity-correlated partial Spearman’s correlation and statistical significance.

### Immune Cell Infiltration With the CIBERSORT Algorithm

CIBERSORT (https://cibersort.stanford.edu/), an established computational resource, was applied to characterize the immune cell composition based on a validated leukocyte gene signature matrix containing 547 genes and 22 human immune cell subpopulations. Our current analysis gauged the proportions of tumor-infiltrating immune cells in lung cancer through CIBERSORT and examined the correlations between hepcidin expression and the immune cell subpopulation. A p-value <0.05 was set as the criterion to select lymphocytes possibly affected by hepcidin expression.

### Kaplan-Meier Plotter Database Analysis

We used KM Plotter (http://kmplot.com), an online database that contains gene expression data and survival information of 3452 clinical lung cancer patients, to analyze the prognostic value of hepcidin in lung cancer. The patient samples were separated into two groups by median expression (high expression and low expression) to analyze the overall survival (OS), progression-free survival (PFS) and postprogression survival (PPS) with hazard ratios (HRs) with 95% confidence intervals (95% CIs) and log-rank p-values.

### PrognoScan Database Analysis

The correlation between hepcidin expression and survival in lung cancer was also analyzed by the PrognoScan database (http://www.abren.net/PrognoScan/). The relationships between hepcidin expression and patient prognosis, such as OS and relapse-free survival (RFS), across a large collection of publicly available cancer microarray datasets can be investigated by using PrognoScan. To select the datasets to be included in this study, the screening parameters were set as follows: “Cancer Type” as lung cancer, “Subtype” as “adenocarcinoma” and “squamous cell carcinoma”. HR with 95% CIs was calculated. The threshold was adjusted to a Cox P-value <0.05.

### Analysis of Hepcidin-Interacting Genes and Proteins

The GeneMANIA database (http://www.genemania.org) was applied to construct the hepcidin interaction network. The STRING online database (https://string-db.org/) was applied to construct a protein-protein interaction (PPI) network of hepcidin.

### Cell Culture, RNA Isolation and Real-Time PCR

The human lung epithelial cell line BEAS-2B and NSCLC cell lines HCC827 and A549 were cultured in Dulbecco’s modified Eagle’s medium (DMEM, Gibco) containing 10% heat-inactivated fetal bovine serum (FBS) and 1% penicillin/streptomycin. All cells were incubated in an incubator with 5% CO^2^ at 37°C. Real-time PCR was conducted to evaluate gene expression. Total RNA was extracted from fresh renal tissues or cells using a TRIzol-based method as previously described (39, 40). Real-time PCR was performed in triplicate using samples derived from three independent experiments. Primers for hepcidin (forward, 5’- CTGACCAGTGGCTCTGTTTTCC-3’, reverse, 5’- AAGTGGGTGTCTCGCCTCCTTC-3’) and S18 (forward, 5’-GTTCCGACCATAAACGATGCC-3’, reverse, 5’-TGGTGGTGCCCTTCCGTCAAT-3’) were used for qPCR.

### Immunohistochemistry (IHC) Staining

This study was approved by the Institutional Research Ethics Committee of HanDan Central Hospital. Written informed consent was obtained from the participants. Ten formalin-fixed, paraffin-embedded lung cancer tissues and normal lung tissues were used for IHC staining. Briefly, 4-μm sections of tissues were mounted on glass microscope slides, deparaffinized in xylene, and then rehydrated in sequentially increasing dilutions of alcohol. Antigen retrieval was performed at a high temperature using a water bath. The sections were cooled and rinsed, and endogenous peroxidases were quenched using 3% hydrogen peroxide. Then, the sections were washed three times with PBS, incubated with calf serum to block nonspecific antigens for 10 min, incubated with anti-hepcidin polyclonal primary antibody (1:200, ab30760, Abcam, Cambridge, MA, USA) overnight at 4°C, washed with PBS three times, and then incubated with secondary antibody for 30-40 min at room temperature (RT). Dried sections were observed with an optical microscope. The IHC staining results were analyzed and scored by two pathologists who were blinded to the sources of the clinical samples. A semiquantitative integration method was used to analyze the intensity of staining.

### Statistical Analysis

The results generated in Oncomine are displayed with P-values, fold changes, and ranks. The results of Kaplan-Meier plots, PrognoScan, and GEPIA are displayed with HR and P or Cox P-values from a log-rank test. The correlation of gene expression was evaluated by Spearman’s correlation and statistical significance. The heat map of the correlations between hepcidin and iron metabolism-related genes was generated by the R software package pheatmap with Spearman’s correlation. P-values < 0.05 were considered statistically significant.

## Results

### Hepcidin Expression Is Increased in Lung Cancer Patients

**Figure 1:**
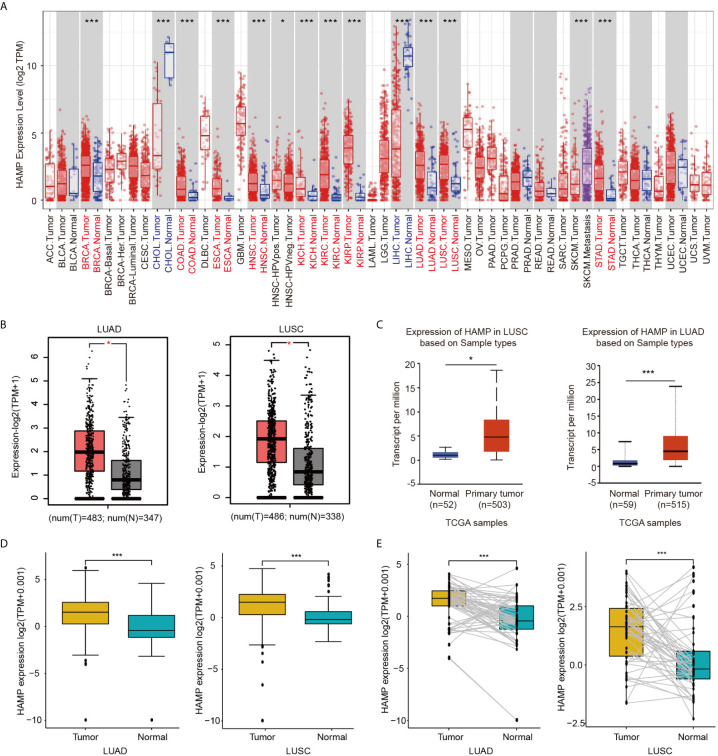
Expression of hepcidin in lung cancer. **(A)** Hepcidin expression in different types of cancer was investigated with the TIMER database. **(B)** Increased or decreased expression of hepcidin in lung cancer compared to normal tissues in the GEPIA database. **(C)** Hepcidin expression in lung cancer was examined by using the UALCAN database. **(D)** Analysis of hepcidin expression in lung cancer and adjacent normal tissues in the TCGA database. **(E)** TCGA database and statistical analyses of hepcidin expression in 58 pairs of LUAD tissues and adjacent normal tissues and 50 pairs of LUSC tissues and adjacent normal tissues, respectively. *p < 0.05, ***p < 0.001.

The mRNA expression of hepcidin in human cancers was first analyzed using the Tumor Immune Estimation Resource (TIMER) online database. Higher expression of hepcidin was observed in breast invasive carcinoma (BRCA), colon adenocarcinoma (COAD), esophageal carcinoma (ESCA), head and neck squamous cell carcinoma (HNSC), kidney chromophobe (KICH), kidney renal papillary cell carcinoma (KIRP), LUAD, LUSC and stomach adenocarcinoma (STAD) compared with the corresponding normal tissues (**Figure 1A**). Consistently, we also found that higher mRNA of hepcidin was expressed in LUAD and LUSC tissues than in normal lung tissues in the gene expression profiling interactive analysis (GEPIA) and UALCAN databases (**Figures 1B, C**). The expression of hepcidin mRNA was further examined using the Oncomine database (**Supplementary Figure 1**). We found that hepcidin expression was higher in LUAD, LUSC and LCC tissues from 5 different cohorts (**Supplementary Figure 1**). In addition, hepcidin expression in LUAD and LUSC samples and adjacent normal tissues was analyzed using data directly obtained from The Cancer Genome Atlas (TCGA). Hepcidin expression was significantly elevated in LUAD and LUSC tissues (**Figure 1D**). Furthermore, a marked increase in hepcidin expression in LUAD and LUSC was observed in 58 and 50 paired tumor samples compared with adjacent normal samples, respectively (**Figure 1E**). These findings illustrate that hepcidin expression is upregulated in lung cancer and indicate that hepcidin may play an important regulatory role in lung cancer progression.

**Figure 2:**
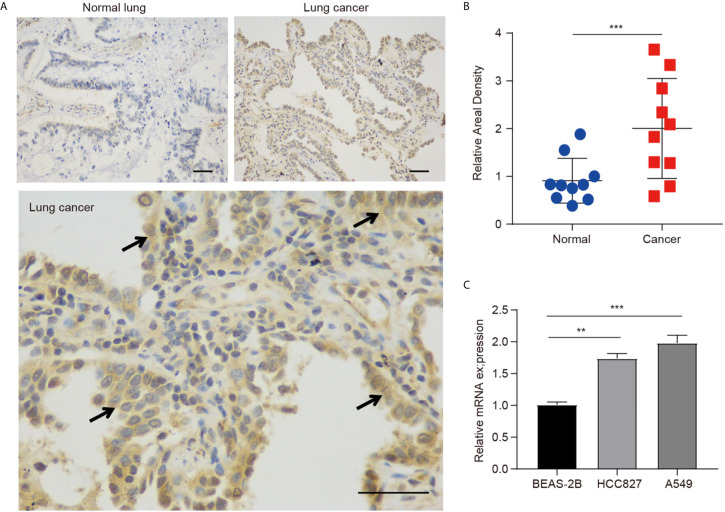
Protein expression of hepcidin in lung cancer patients. **(A)** Immunohistochemical staining of hepcidin was performed in lung cancer and normal lung tissues. Representative images are shown. Scare bars, 50 μM. **(B)** The staining was quantified, as shown. The dot plot depicts the means and standard deviation of 10 images of lung cancer patient tissues and normal lung tissues. **(C)** Hepcidin expression in three different cell lines was examined by real-time PCR. The mean ± s.d. is shown. Statistical significance was determined using one-way ANOVA with the post hoc Tukey test. **p < 0.01, ***p < 0.001.

The protein expression of hepcidin was further investigated in lung cancer by IHC staining, and we found that the hepcidin protein level was obviously increased in lung cancer tissues compared with normal lung tissues (**Figures 2A, B**). Moreover, we found that hepcidin mRNA expression was significantly upregulated in two NSCLC cell lines (A549 and HCC827) compared to that in a nonmalignant lung epithelial cell line (BEAS-2B) (**Figure 2C**).

### Hepcidin Expression and Clinical Parameters of Lung Cancer Patients

**Figure 3:**
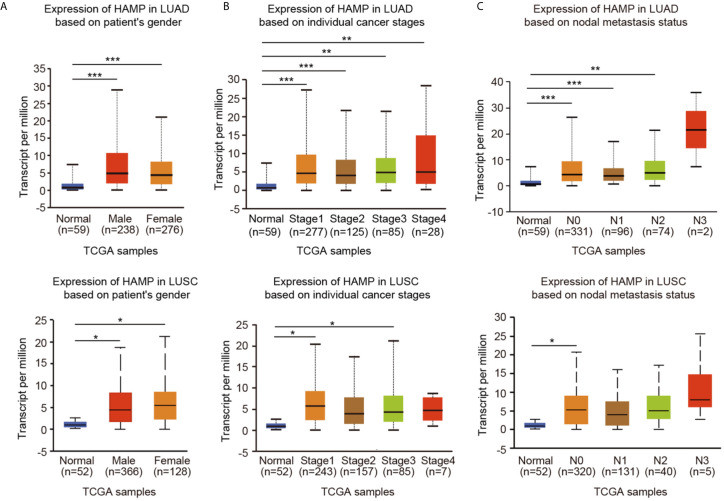
Box plots evaluating hepcidin expression among different groups of patients based on clinical parameters using the UALCAN database. Analysis is shown for sex **(A)**, cancer stage **(B)**, and metastasis **(C)**. N0: no regional lymph node metastasis; N1: metastases in 1 to 3 axillary lymph nodes; N2: metastases in 4 to 9 axillary lymph nodes; N3: metastases in 10 or more axillary lymph nodes. *p < 0.05, **p < 0.01, ***p < 0.001.

By using the UALCAN online tool, we then investigated hepcidin expression among groups of patients according to different clinical parameters. According to sex, hepcidin expression was significantly upregulated in lung cancer samples from both males and females compared to the corresponding normal controls (**Figure 3A**). Regarding tumor stage, a significant increase in hepcidin expression was observed in LUAD patients in stages 1, 2, 3 and 4 and in LUSC patients in stages 1 and 3 (**Figure 3B**). Based on cancer stage, hepcidin expression was higher in patients with LUAD classified as N0, N1 or N2 and in patients with LUSC classified as N0 (**Figure 3C**). Upregulation of hepcidin expression was observed in both TP53-mutant and TP53 wild-type lung cancer patients compared to normal controls (**Supplementary Figure 2A**). In terms of age, the hepcidin level was significantly elevated in the lung cancer tissues of patients from different age groups (21-40 years, 41-60 years, 61-80 years and 81-100 years in LUAD; 61-80 years in LUSC) (**Supplementary Figure 2B**). In addition, hepcidin expression was dramatically increased in Caucasian lung cancer patients (**Supplementary Figure 2C**). These results suggest that there is a close correlation between hepcidin expression and tumor progression and metastasis.

### Increased Hepcidin Expression Correlates With Poor Prognosis in Lung Cancer Patients

**Figure 4:**
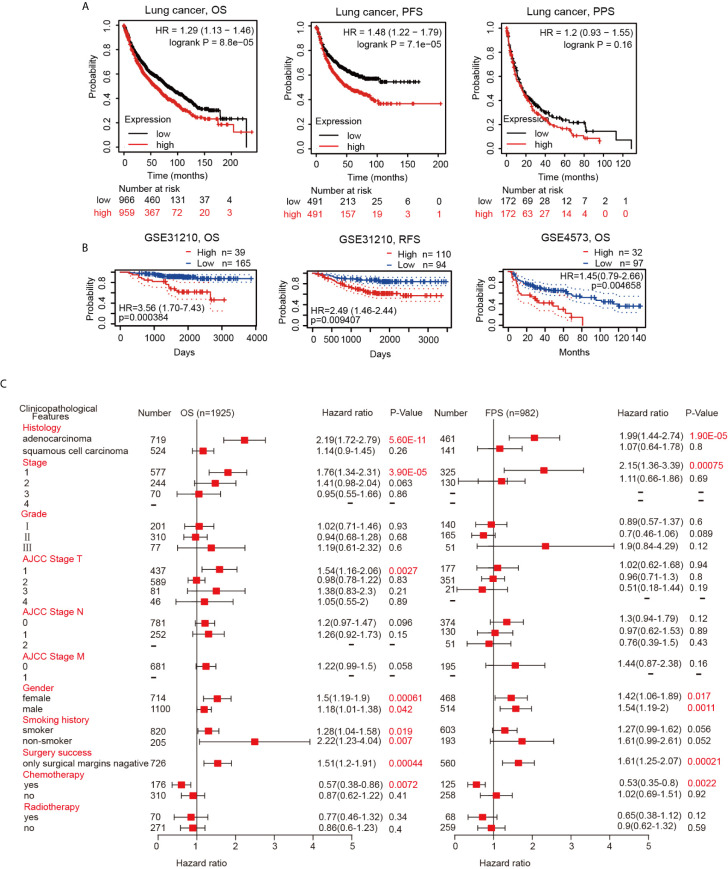
Survival curve evaluating the prognostic value of hepcidin. **(A)** Survival curves using the Kaplan-Meier plotter are shown for OS, PFS and PPS. **(B)** Survival curves using the PrognoScan database are shown for OS and RFS. **(C)** A forest plot shows the correlation between hepcidin expression and clinicopathological parameters in LUAD and LUSC patients.

Since the hepcidin expression level is intimately related to lung cancer progression and metastasis, we then examined the prognostic value of the hepcidin gene. Lung cancer patients with higher expression of the hepcidin gene exhibited poor overall survival (OS) and progression-free survival (PFS) but not postprogression survival (PPS) according to the Kaplan-Meier plotter database (**Figure 4A**). Moreover, the PrognoScan database demonstrated that elevated expression of hepcidin was significantly associated with poor OS and RFS in the GSE31210 and GSE4573 cohorts (**Figure 4B**). These results indicate that hepcidin is significantly associated with the prognosis of lung cancer patients.

### Validation of the Prognostic Value of Hepcidin Based on Various Clinicopathological Features

To better understand the prognostic value and potential mechanism of hepcidin expression in lung cancer, we explored the association between hepcidin mRNA expression and clinical characteristics using the Kaplan-Meier database. Interestingly, hepcidin upregulation was correlated with poor OS and poor PFS in LUAD patients but not in LUSC patients (**Figure 4C**). High hepcidin expression was significantly correlated with poor OS and PFS in male and female lung cancer patients (**Figure 4C**). Regarding different tumor stages, high hepcidin expression was associated with poor OS and poor PFS only in stage 1 lung cancer patients (**Figure 4C**). A significant correlation between hepcidin expression and poor OS was observed in American Joint Committee on Cancer (AJCC) stage T-1 lung cancer patients (**Figure 3C**). Moreover, we found a significant association between hepcidin expression and unfavorable OS in both smoking and nonsmoking lung cancer patients (**Figure 4C**). In addition, high hepcidin expression was significantly associated with poor OS and PFS in lung cancer patients with negative surgical margins (**Figure 4C**). In contrast, upregulated hepcidin levels corresponded with better OS and PFS in patients with chemotherapy (**Figure 4C**). These results imply that hepcidin mRNA expression possesses prognostic value in lung cancer.

### Identification of Hepcidin-Interacting Genes and Proteins and Genetic Alterations

**Figure 5:**
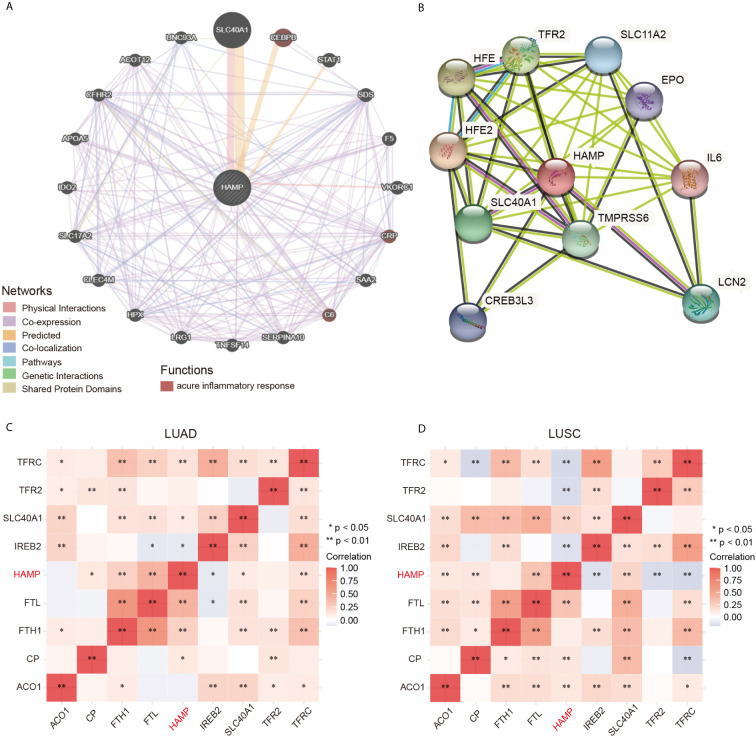
**(A)** The gene-gene interaction network of hepcidin was constructed using GeneMania. **(B)** The PPI network of hepcidin was generated using STRING. **(C, D)** A heat map shows the correlations between hepcidin and iron metabolism-related genes in LUAD and LUSC, respectively. *p < 0.05, **p < 0.01.

We constructed the gene-gene interaction network for hepcidin and the altered neighboring genes by using GeneMania. The results showed that the 20 most frequently altered genes were closely correlated with hepcidin, including SLC40A1, CEBPB, and STAT1 (**Figure 5A**). Functional analysis suggested that these genes were significantly associated with the acute inflammatory response (**Figure 5A**). A protein-protein interaction (PPI) network of hepcidin was generated using the STRING database (**Figure 5B**). There were 43 edges and 11 nodes, including SLC40A1, TFR2 and HFE (**Figure 5B**). In addition, the correlations between hepcidin and iron metabolism-related genes were investigated based on TCGA database. Hepcidin was positively and significantly correlated with CP, FTH1, FTL, SLC40A1 and TFRC but negatively correlated with TFR2 IREB2 in LUAD (**Figure 5C**). Moreover, hepcidin was positively and significantly correlated with ACO1, CP, FTL and SLC40A1 but negatively correlated with IREB2, TFR2 and TFRC in LUSC (**Figure 5D**).

The alteration frequency of hepcidin in lung cancer was analyzed using cBioPortal. A total of 2197 patients from three datasets of lung cancer were analyzed (NSCLC, TCGA, 2016; LUSC, TCGA, pancancer altas; LUAD, TCGA, pancancer altas). Genetic variations in hepcidin showed incidence rates of 5.59%, 5.34%, and 3.18% in these three datasets, respectively (**Supplementary Figures 3A, B**). Amplification was the most common type (**Supplementary Figures 3A, B**). However, the results of Kaplan–Meier plotter and log-rank test indicated that there was no statistically significant difference between OS and PFS and lung cancer patients with or without alterations of hepcidin (**Supplementary Figure 3C**).

### Gene Ontology (GO) and Kyoto Encyclopedia of Genes and Genomes (KEGG) Pathway Analysis of Hepcidin and Its Coexpressed Genes in TCGA Lung Cancer

**Figure 6:**
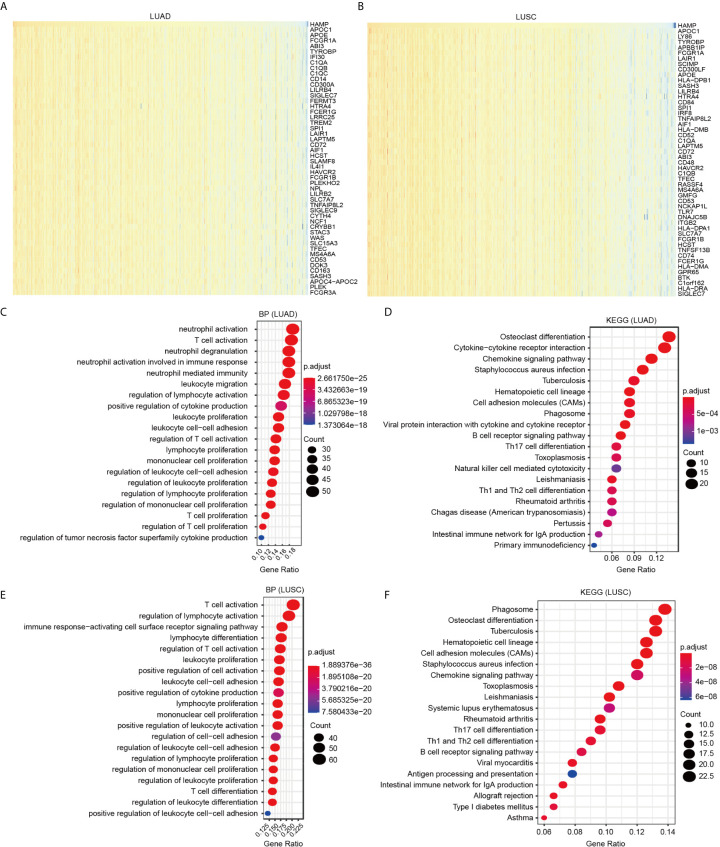
GO and KEGG enrichment analysis for hepcidin. **(A)** Heat maps showing the top 50 genes positively correlated with hepcidin in LUAD. **(B)** Heat maps showing the top 50 genes positively correlated with hepcidin in LUSC. **(C)** Top 20 enrichment terms in BP categories in LUAD. **(D)** Top 20 enrichment terms in BP categories in LUSC. **(E)** Top 20 KEGG enrichment pathways in LUAD. **(F)** Top 20 KEGG enrichment pathways in LUSC.

Data mining from TCGA database was used to identify genes positively or negatively coexpressed with hepcidin. The top 50 genes that were positively and negatively correlated with hepcidin in LUAD and LUSC are shown (**Figures 6A, B**; **Figures S4A, B**). Then, a total of 300 genes positively related to hepcidin were used for KEGG and GO enrichment analyses to explore the hepcidin-related pathways and biological functions. The top 20 significant terms of BP, MF and CC enrichment analysis are presented (**Figure 6**; **Supplementary Figures 4C–F**). Notably, in terms of BP, hepcidin was enriched in immune response-related processes, such as neutrophil activation, T cell activation, leukocyte proliferation and migration, and positive regulation of cytokine production in LUAD; the enriched processes in LUSC were T cell activation, regulation of lymphocyte activation, immune response-activating cell surface receptor signaling pathway, lymphocyte differentiation, leukocyte proliferation, etc. (**Figures 6C, E**).

In addition, the top 20 KEGG pathways for hepcidin and its-correlated genes are shown in **Figures 6D, F**. Among these pathways, many immune-related pathways were highly associated with hepcidin, including cytokine-cytokine receptor interaction, chemokine signaling pathway, B cell receptor signaling pathway, Th17 cell differentiation, natural killer cell-mediated cytotoxicity, Th1 and Th2 cell differentiation and intestinal immune network for IgA production in LUAD; and chemokine signaling pathway, Th17 cell differentiation, Th1 and Th2 cell differentiation, B cell receptor signaling pathway, antigen processing and presentation, and intestinal immune network for IgA production in LUSC (**Figures 6D, F**).

### Gene Set Enrichment Analysis (GSEA) Identified Hepcidin-Related Signaling Pathways

To further explore the molecular mechanisms affected by hepcidin in lung cancer, GSEA was conducted. Among the GO terms, the top 20 signaling pathways influenced by hepcidin were enriched mainly in immune-related activities, including adaptive immune response, immune effector process, activation of immune response, cytokine production, activation of innate immune response, and regulation of cytokine-mediated signaling pathway in LUAD; and adaptive immune response, leukocyte mediated immunity, cell activation involved in immune response, cytokine production, myeloid cell activation involved in immune response and neutrophil activation involved in immune response in LUSC (**Supplementary Figures 5A, B**). Similarly, among the KEGG terms, GSEA revealed multiple immune functional gene sets that were enriched in lung cancer, including those related to viral protein interactions with cytokine and cytokine receptors, natural killer cell-mediated cytotoxicity, cytokine-cytokine receptor interaction and chemokine signaling pathways (**Supplementary Figures 5C, D**). These results strongly imply that hepcidin is involved in the regulation of the immune response in lung cancer.

### Correlation Analysis Between Hepcidin Expression and Infiltrating Immune Cells

**Figure 7:**
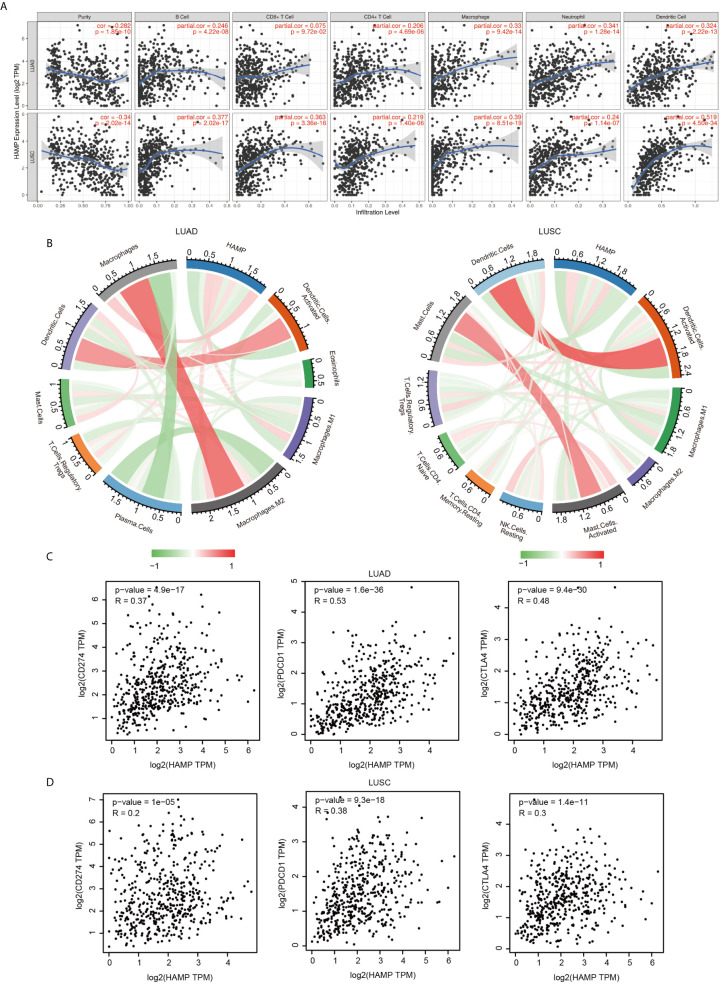
Correlation of hepcidin expression with immune infiltration level. **(A)** Hepcidin is significantly associated with tumor purity and is positively correlated with the infiltration of different immune cells using the TIMER database. **(B)** Hepcidin expression has a significant correlation with the infiltration of immune cells in lung cancer using the CIBERSORT algorithm. **(C, D)** Scatterplots of the correlations between hepcidin expression and PD-1, PD-L1 and CTLA-4 in LUAD and LUSC using the GEPIA database.

We analyzed the correlation between hepcidin expression and six types of infiltrating immune cells, including B cells, CD4+ T cells, CD8+ T cells, neutrophils, macrophages, and dendritic cells. The results showed that hepcidin expression levels had a significant positive correlation with the infiltration of B cells, CD4+ T cells, macrophages, neutrophils, and dendritic cells and no significant correlations with CD8+ T cells in LUAD (**Figure 7A**). Moreover, hepcidin expression was positively and significantly associated with infiltration of all six types of immune cells in LUSC (**Figure 7A**).

To further assess the effect of hepcidin on the tumor microenvironment (TME), we estimated the correlation between hepcidin and immune infiltration using the established computational resource CIBERSORT. Notably, hepcidin was positively correlated with the infiltration levels of macrophages, M1 macrophages, M2 macrophages and regulatory Treg cells but negatively correlated with the infiltration levels of dendritic cells, activated dendritic cells, mast cells, resting mast cells, monocytes, plasma cells, lymphocytes, and eosinophils in LUAD (**Figure 7B**; **Supplementary Figure 6**). Moreover, hepcidin was positively correlated with the infiltration levels of CD4 memory T cells, gamma delta T cells, regulatory Treg cells, activated memory CD4 T cells, macrophages, M1 macrophages and M2 macrophages but negatively correlated with the infiltration levels of naïve CD4 T cells, mast cells, activated mast cells, dendritic cells, activated dendritic cells, eosinophils, and M0 macrophages in LUSC (**Figure 7B**; **Supplementary Figure 7**).

### Correlation Between Hepcidin Expression and Various Immune Markers

To deepen our understanding of hepcidin crosstalk with the immune response, we validated the correlations between hepcidin expression and diverse immune signatures in both LUAD and LUSC using the TIMER database. The genes listed in **Table 1** were used to characterize immune cells, including B cells, T cells, CD8+ T cells, monocytes, tumor-associating macrophages (TAMs), M1 macrophages, M2 macrophages, neutrophils, natural killer (NK) cells and dendritic cells. Tumor purity is an important aspect affecting the dissection of immune infiltration in clinical cancer biopsies. After adjusting for tumor purity, hepcidin expression was significantly associated with most immune markers in divergent types of immune cells in LUSC and LUAD (**Table 1**).

**Table 1:** Correlation analysis between hepcidin and gene markers of immune cells in TIMER.

Description	Gene markers	LUAD				LUSC			
		None		Purity		None		Purity	
		Cor	p	Cor	p	Cor	p	Cor	p
**B cell**	CD19	0.285	***	0.191	***	0.463	***	0.36	***
	CD79A	0.25	***	0.156	***	0.457	***	0.354	***
**T cell (general)**	CD3D	0.358	***	0.27	***	0.58	***	0.508	***
	CD3E	0.354	***	0.262	***	0.586	***	0.517	***
	CD2	0.394	***	0.313	***	0.61	***	0.55	***
**CD8+ T cell**	CD8A	0.337	***	0.247	***	0.517	***	0.463	***
	CD8B	0.318	***	0.249	***	0.4	***	0.376	***
**Monocyte**	CD86	0.482	***	0.421	***	0.574	***	0.501	***
	CSF1R	0.442	***	0.383	***	0.576	***	0.499	***
**TAM**	CCL2	0.358	***	0.295	***	0.436	***	0.366	***
	CD68	0.468	***	0.405	***	0.492	***	0.424	***
	IL10	0.467	***	0.392	***	0.46	***	0.393	***
**M1**	IRF5	0.32	***	0.257	***	0.213	***	0.19	***
	PTGS2	-0.153	***	-0.171	***	0.182	***	0.265	**
	NOS2	0.201	***	0.152	***	0.068	0.129	0.096	*
**M2**	CD163	0.416	***	0.346	***	0.553	***	0.486	***
	VSIG4	0.444	***	0.385	***	0.59	***	0.534	***
	MS4A4A	0.476	***	0.413	***	0.633	***	0.579	***
**Neutrophils**	CEACAM8	-0.05	0.260	-0.073	0.105	0.084	0.0588	0.074	0.108
	ITGAM	0.348	***	0.283	***	0.481	***	0.398	***
	CCR7	0.274	***	0.164	***	0.496	***	0.413	***
**Natural killer cell**	KIR2DL1	0.177	***	0.148	***	0.201	***	0.163	***
	KIR2DL3	0.217	***	0.161	***	0.273	***	0.227	***
	KIR2DL4	0.265	***	0.214	***	0.232	***	0.169	***
	KIR3DL1	0.218	***	0.185	***	0.335	***	0.289	***
	KIR3DL2	0.206	***	0.15	***	0.327	***	0.286	***
	KIR3DL3	0.12	**	0.114	*	0.119	**	0.118	**
	KIR2DS4	0.208	***	0.17	***	0.24	***	0.216	***
**Dendritic cell**	HLA-DPB1	0.319	***	0.241	***	0.682	***	0.627	***
	HLADQB1	0.29	***	0.219	***	0.526	***	0.453	***
	HLA-DRA	0.315	***	0.235	***	0.649	***	0.588	***
	HLA-DPA1	0.294	***	0.216	***	0.657	***	0.597	***
	CD1C	0.085	0.0551	0.015	0.735	0.423	***	0.298	***
	NRP1	-0.045	0.308	-0.074	0.0986	0.261	***	0.158	***
	ITGAX	0.474	***	0.413	***	0.557	***	0.477	***

*p < 0.05, **p < 0.01, ***p < 0.001.

We also examined the correlation between hepcidin expression and various functional T cells, including Th1, Th1-like, Th2, Treg, resting Tregs, effector Tregs, effector T cells, naïve T cells, effector memory T cells, resistant memory T cells, and exhausted T cells (**Table 2**). By using the TIMER database, we found that the hepcidin expression level was significantly correlated with 33 of 38 T cell markers in LUAD and with 32 of 38 T cell markers in LUSC after adjusting for tumor purity (**Table 2**).

**Table 2:** Correlation analysis between hepcidin and gene markers of different types of T cells in TIMER.

Description	Gene markers	LUAD				LUSC			
		None		Purity		None		Purity	
		Cor	p	Cor	p	Cor	p	Cor	p
Th1	TBX21	0.336	***	0.243	***	0.53	***	0.46	***
	STAT4	0.24	***	0.136	**	0.469	***	0.38	***
	STAT1	0.272	***	0.196	***	0.241	***	0.194	***
	TNF	0.215	***	0.126	**	0.17	***	0.049	0.285
	IFNG	0.352	***	0.284	***	0.389	***	0.346	***
Th1-like	HAVCR2	0.546	***	0.495	***	0.681	***	0.63	***
	IFNG	0.352	***	0.284	***	0.389	***	0.346	***
	CXCR3	0.383	***	0.301	***	0.615	***	0.559	***
	BHLHE40	-0.007	0.879	-0.029	0.514	-0.005	0.920	-0.076	0.0954
	CD4	0.426	***	0.355	***	0.637	***	0.577	***
Th2	STAT6	-0.085	0.0535	-0.097	*	-0.071	0.112	-0.075	0.101
	STAT5A	0.316	***	0.236	***	0.404	***	0.314	***
Treg	FOXP3	0.298	***	0.208	***	0.49	***	0.403	***
	CCR8	0.209	***	0.117	**	0.444	***	0.365	***
	TGFB1	0.165	***	0.089	*	0.055	0.221	-0.044	0.333
Resting Treg	FOXP3	0.298	***	0.208	***	0.49	***	0.403	***
	IL2RA	0.308	***	0.23	***	0.505	***	0.431	***
Effector Treg T-cell	FOXP3	0.298	***	0.208	***	0.49	***	0.403	***
	CCR8	0.209	***	0.117	**	0.444	***	0.365	***
	TNFRSF9	0.318	***	0.23	***	0.431	***	0.344	***
Effector T-cell	CX3CR1	0.239	***	0.2	***	0.481	***	0.402	***
	FGFBP2	0.184	***	0.145	**	-0.017	0.702	0.025	0.579
	FCGR3A	0.517	***	0.461	***	0.604	***	0.545	***
Naïve T-cell	CCR7	0.274	***	0.164	***	0.496	***	0.413	***
	SELL	0.3	***	0.205	***	0.508	***	0.422	***
Effector memory T-cell	DUSP4	0.072	0.101	0.068	0.131	0.294	***	0.232	***
	GZMK	0.328	***	0.242	***	0.582	***	0.518	***
	GZMA	0.409	***	0.341	***	0.461	***	0.395	***
Resident memory T-cell	CD69	0.24	***	0.14	**	0.5	***	0.413	***
	CXCR6	0.371	***	0.287	***	0.587	***	0.531	***
	MYADM	0.118	**	0.042	0.350	0.299	***	0.215	***
General memory T-cell	CCR7	0.274	***	0.164	***	0.496	***	0.413	***
	SELL	0.3	***	0.205	***	0.508	***	0.422	***
	IL7R	0.211	***	0.106	0.0181	0.331	***	0.215	***
Exhausted T-cell	HAVCR2	0.546	***	0.495	***	0.681	***	0.63	***
	LAG3	0.368	***	0.305	***	0.417	***	0.359	***
	CXCL13	0.259	***	0.16	***	0.361	***	0.263	***
	LAYN	0.17	***	0.077	0.0886	-0.002	0.971	-0.005	0.919

*p < 0.05, **p < 0.01, ***p < 0.001.

We further investigated the interrelationship between hepcidin expression and famous T cell checkpoints, such as PD-1, PD-L1 and CTLA-4, in the GEPIA database. Hepcidin expression was significantly correlated with the expression of PD-1, PD-L1 and CTLA-4 in LUAD and LUSC (**Figures 7C, D**). These findings further support that hepcidin expression is significantly related to immune infiltration and suggest that hepcidin plays an important role in immune escape in the lung cancer microenvironment.

### Prognostic Analysis of Hepcidin Expression Based on Immune Cells in LUSC Patients

**Figure 8:**
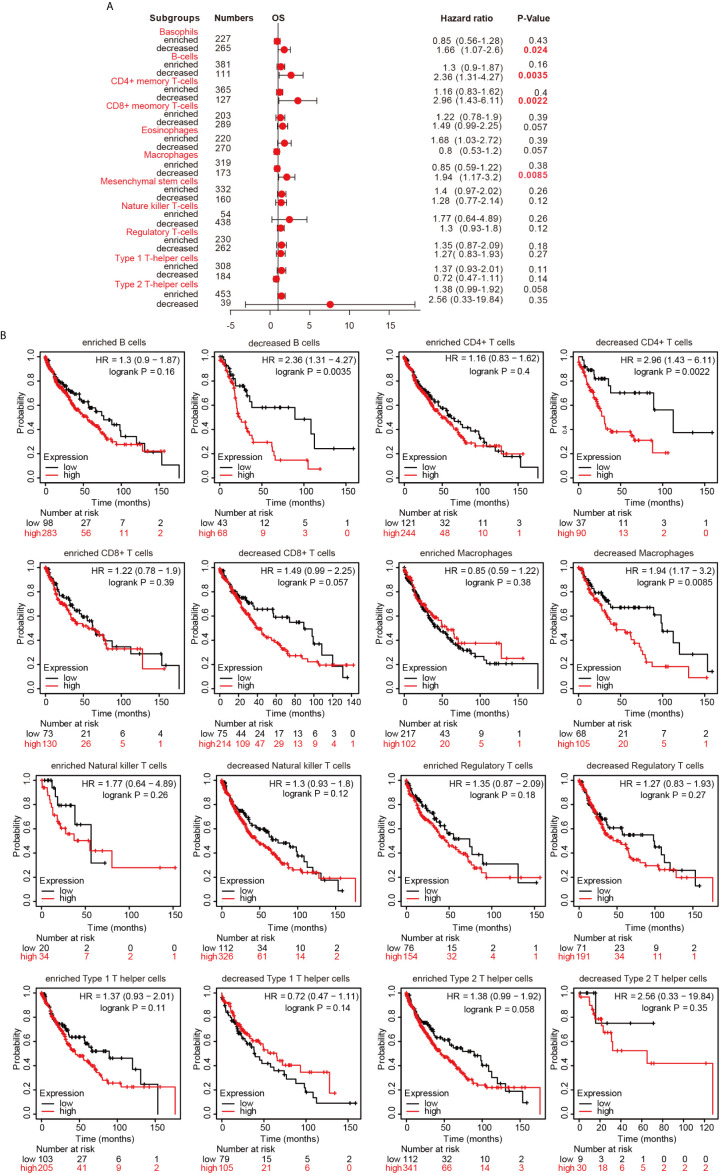
Kaplan-Meier survival curves according to high and low expression of hepcidin in immune cell subgroups in lung cancer. **(A)** A forest plot shows the prognostic value of hepcidin expression according to different immune cell subgroups in LUSC patients. **(B)** Correlations between hepcidin expression and OS in different immune cell subgroups in LUSC patients were estimated by Kaplan-Meier plotter.

Since hepcidin expression is significantly correlated with immune infiltration and poor prognosis in LUSC, we investigated whether hepcidin expression affects the prognosis of LUSC because of immune infiltration. We performed prognosis analyses based on the expression levels of hepcidin in LUSC in related immune cell subgroups. As shown in **Figures 8A, B**, LUSC patients with high expression of hepcidin and decreased infiltration of B cells, CD4+ memory T cells, macrophages and basophils had a poor prognosis. However, there was no significant correlation between hepcidin expression and the prognosis of LUSC in the group with different levels of CD8+, NK, Treg, Th1 and Th2 cell infiltration (**Figures 8A, B**). These results indicate that hepcidin may affect the prognosis of LUSC patients in part due to immune infiltration.

## Discussion

Among malignancies, lung cancer has the highest morbidity rates and is the leading cause of cancer-related death in both males and females worldwide (1). Despite advances in early diagnosis and targeted and immune therapies, lung cancer is often diagnosed at an advanced stage and has a poor prognosis (1–3). Thus, it is important to explore mechanisms that result in the incidence of lung cancer metastasis and identify useful prognostic biomarkers of lung cancer. In the present study, we showed that the expression of hepcidin in lung cancer was higher than that in normal lung tissue by means of bioinformatics analysis of the TIMER, Oncomine, UALCAN and TCGA public databases (**Figure 1**). These findings were consistent with a previous report and suggested that hepcidin may act as an oncogene by promoting the development and progression of lung cancer (34). Subsequently, the clinical prognostic significance of hepcidin in lung cancer patients was investigated. High expression of hepcidin was significantly correlated with sex, age, clinical stage, histological grade and metastasis in lung cancer patients (**Figure 3**). According to the patient samples in the cBioPortal database, approximately 5% of lung cancer patients possess genetic alterations in hepcidin (**Supplementary Figure 3**). We also unearthed the fact that most of the alterations of hepcidin are gene amplifications in lung cancer patients. Furthermore, Kaplan-Meier survival analyses indicated that lung cancer patients with high hepcidin expression exhibited a markedly worse survival rate than those with low expression (**Figure 4**). These results substantiated that hepcidin may be an independent prognostic biomarker in lung cancer and may facilitate the development of targeted precision oncology.

As the most common subtype of NSCLC, accumulating evidence has demonstrated that LUAD and LUSC differ from each other in their biopathology, molecular, clinical characteristics and therapeutic effect (41). For example, the subtypes of LUSC include primitive, classical, secretory and basal (42). Three distinct subtypes of LUAD were introduced in 2014, including proximal inflammatory (PI), proximal proliferative (PP), and terminal respiratory unit (TRU) (43). LUAD usually arises from the distal airway, while LUSC is associated with more proximal airways (44). LUSC is generally more strongly associated with smoking and inflammatory diseases than LUAD (44). In general, LUAD grows more slowly and has smaller lumps than its contemporaneous counterpart LUSC but tends to metastasize at an early stage (45). The most commonly mutated genes in LUAD include oncogenes (KRAS and EGFR) and tumor suppressor genes (TP53, KEAP1, STK11 and NF1) (46). The frequency of EGFR-activating mutations varies greatly by region and ethnicity. In contrast, the commonly mutated gene in LUSC is TP53, which is observed in more than 80% of the samples. Recurrent mutations in NFE2L2, KEAP1, CDKN2A, FBXW7, BAI3, GRM8, MUC16, RUNX1T1, STK11 and ERBB4 have been reported in LUSC (46). Moreover, many studies have investigated the differences in the mRNA and circRNA expression profiles and methylation patterns of LUAD and LUSC. These findings provide more insights into the molecular mechanism of LUSC and LUAD. Consistent with these observations in LUAD and LUSC, we also found that there were some differences in our analysis results between LUAD and LUSC. For instance, upregulated hepcidin expression only significantly correlated with poor OS and PFS in LUAD but not in LUSC (**Figure 4**). Moreover, there were few overlapping enrichment terms in GO and KEGG analyses between LUAD and LUSC. Nevertheless, we still found that hepcidin was closely associated with immune response-related pathways in both LUAD and LUSC (**Figure 6** and **Supplementary Figure 5**).

Human hepcidin is highly expressed in hepatocytes. In addition to the liver, hepcidin is also synthesized in a number of other organs and tissues, such as the brain, heart, kidney, spleen, pancreas, stomach and adipose tissue (47). The function of this extrahepatic hepcidin remains unclear, but one hypothesis is that it is associated with local iron homeostasis. A growing number of studies have suggested that increased serum hepcidin accompanies multiple cancers, including breast cancer, prostate cancer, renal cell carcinoma and myeloma (23). Furthermore, recent studies have revealed that hepcidin can be produced by cancer cells. For example, hepcidin expression was observed in normal breast cells and was significantly increased in breast cancer cells (25, 48). Suppression of hepcidin synthesis by heparin, a potent inhibitor of liver-derived hepcidin production, induced significant inhibition of tumor growth due to diminished intracellular iron retention (48). These results imply that circulating hepcidin secreted from the liver exerts a robust effect on tumor growth by mediating ferroportin-regulated iron export in cancer cells. More importantly, knockdown of tumor hepcidin expression also caused robust inhibition of tumor growth of MDA-MB-231 cells (48). Hepcidin was also highly expressed in prostate cancer cells compared with normal prostate epithelial cells (32, 49). Hepcidin synthesis in prostate cancer cells is regulated by Wnt- and SOSTDC1-associated pathways (32, 49). Inhibition of hepcidin obviously suppressed prostate cancer cell survival. Hepcidin could be detected in pancreatic cancer and gastric cancer tissues by IHC staining (30, 50). Strongly stained hepcidin patients showed a worse OS than weakly stained hepcidin patients with pancreatic cancer (30). Furthermore, hepcidin expression was significantly increased in thyroid cancer cells, especially K1 and 8505C cells, compared with normal cells (51). Mechanistically, SOSTDC1 silencing by E4BP4 and G9a complex-mediated promoter hypermethylation promoted hepcidin secretion in thyroid cancer (51). In addition, knockout of *hepcidin* led to a marked reduction in the development of cancer in a mouse lung cancer model (52). A previous study demonstrated that hepcidin expression in doxorubicin-resistant MCF-7 cells was increased compared with that in doxorubicin-sensitive MCF-7 cells (27). Moreover, the development of resistance to doxorubicin in Walker-256 carcinosarcoma *in vivo* was accompanied by an increase in hepcidin expression (27). However, the underlying mechanism between hepcidin and chemoresistance is still unclear. A possible explanation is the upregulation of IL-6 expression and consequent upregulation of hepcidin associated with inflammatory conditions typically observed in many patients with metastatic cancer. Increased IL-6 concentrations have been demonstrated to be closely associated with chemoresistance. In esophageal squamous cell carcinoma, IL-6 derived from cancer-associated fibroblasts plays the most important role in chemoresistance by upregulating the expression of C-X-C motif chemokine receptor 7 (CXCR7) *via* the STAT3/nuclear factor-κB (NF-kB) pathway (53). IL-6 contributes to chemoresistance in MDA-MB-231 cells by upregulating HIF-1α through the activation of STAT3 (54). In addition, IL-6 also enhanced the chemoresistance of ovarian cancer cells against cisplatin through the IL-6/STAT3/HIF-1α loop *in vitro* and *in vivo* (55). Hepcidin has been considered a particularly attractive target, and agents that inhibit hepcidin are under active investigation as potential therapies for cancer treatment. Here, we found that hepcidin expression was upregulated in lung cancer tissues compared with normal lung tissues (**Figure 2A**). The expression of hepcidin in A549 and HCC827 cells was also higher than that in normal lung cells (**Figure 2C**). These findings suggest that lung cancer may synthesize functional hepcidin to promote its proliferation. However, the ways and methods to reduce the expression of hepcidin still need to be further explored. Excessive reduction of systemic hepcidin can lead to iron deposition, which is another risk factor for tumor development and progression. Over the last decade, there has been increasing interest in developing pulmonary drug delivery systems suitable for lung cancer therapy (56, 57). A number of nanocarrier systems, including nanoparticles, liposomes, micelles and polymers, have been developed to selectively deliver various anticancer molecules and drugs at the tumor site. Nanocarrier systems have potential advantages, such as improved drug solubility, prolonged systemic circulation, controlled release and targeted drug delivery (56, 57). Moreover, topical delivery of hepcidin-targeted drugs to the lung *via* inhalation is also deemed to be an effective approach for the treatment of lung cancer (58).

Hepcidin is upregulated in response to iron overload (9, 10). Hepcidin is also an acute-phase reactant induced by inflammatory stimuli. A previous study reported that the induction of hepcidin can be triggered by IL-6, which plays an important role in the regulation of inflammation and the immune response (20). However, to our knowledge, the relationship between hepcidin and immune cell infiltration in lung cancer has not been investigated. In the present study, GO and KEGG pathway enrichment analyses of hepcidin and its related genes revealed that hepcidin is involved in numerous pathways, especially the immune system in lung cancer (**Figure 6**). This finding was consistent with the literature and GSEA results we presented in this study, solidifying the association between hepcidin and the immune response (**Supplementary Figure 5**). Here, we first report that high hepcidin expression in lung cancer is correlated with the increased infiltration of B cells, CD4+ T cells, CD8+ T cells, neutrophils, macrophages, and dendritic cells (**Figure 7**). Moreover, a significant association between hepcidin and various immune cell marker sets was observed in lung cancer (**Table 1** and **Table 2**). Hepcidin expression was also positively correlated with PD-1 and CTLA-4 expression (**Figure 7**). More importantly, hepcidin influences the survival time of lung cancer patients partially through immune cell infiltration (**Figure 8**). These findings indicate that hepcidin could be a novel immune-related therapeutic target in lung cancer. However, the precise role of hepcidin in the tumor-immune microenvironment still needs further in-depth exploration.

The present study improves our understanding of the relationship between hepcidin and lung cancer, but some limitations still exist. First, although we investigated the correlation between hepcidin and immune infiltration in LUAD and LUSC patients, there is a lack of interpretation of the immune analysis according to the different subgroups. Second, we observed that hepcidin was strongly expressed in lung cancer cells by IHC analysis. However, the molecular mechanisms and roles of hepcidin in tumor growth, metastasis and immune infiltration and escape need to be explored in further studies. Third, most of the analyses were performed based on mRNA levels of hepcidin in the present study. A deeper analysis, based on protein levels, would make the data more convincing. Fourth, we did not investigate the diagnostic and prognostic value of hepcidin in small cell lung cancer (SCLC) and large cell lung cancer (LCLC) in this study. Overall, our results indicate that hepcidin could serve as a potential novel prognostic biomarker for lung cancer. Moreover, we explored the underlying evidence indicating that hepcidin regulates immune cell infiltration in the TME in lung cancer patients. Therefore, these findings are potentially valuable in advancing our current understanding of not only the role of hepcidin but also its translational use in lung cancer prognosis and immunotherapy.

## Data Availability Statement

The original contributions presented in the study are included in the article/**Supplementary Material**. Further inquiries can be directed to the corresponding author.

## Ethics Statement

The studies involving human participants were reviewed and approved by The Institutional Research Ethics Committee of HanDan Central Hospital. The patients/participants provided their written informed consent to participate in this study.

## Author Contributions

Study concept and design: YF and KT. Acquisition of data: KT, YF, ZS, BL, and FC. Analysis and interpretation of data: KT, ZS, BH, YM, JH, and FC. Statistical analysis: PC, KT, YF, and BH. Drafting of the manuscript: KT. Critical revision and final approval of the manuscript: KT and YC. Obtained funding: YF and KT. Study supervision: KT. All authors contributed to the article and approved the submitted version.

## Funding

This work was partially supported by the China Postdoctoral Science Foundation (2017M621099), the Key Projects of Hebei Normal University (L2018Z07), the Graduate Student Innovation Funding of Hebei Normal University (CXZZSS2021064) and the One Hundred Person Project of Hebei Province (E2016100019).

## Conflict of Interest

The authors declare that the research was conducted in the absence of any commercial or financial relationships that could be constructed as a potential conflict of interest.

## Supplementary Material

Supplementary Figure 1 Box plots comparing hepcidin expression in normal individuals and lung cancer patients obtained from the Oncomine database.

Supplementary Figure 2 Box plots evaluating hepcidin expression among different groups of patients based on clinical parameters using the UALCAN database. Analysis is shown for TP53 mutation status **(A)**, age **(B)**, and race **(C)**. *p < 0.5, **p < 0.01, ***p < 0.001.

Supplementary Figure 3 Alteration frequency of hepcidin. **(A)** Summary of alterations in hepcidin. **(B)** OncoPrint visual summary of alterations in a query of hepcidin from cBioPortal. **(C)** Kaplan-Meier plots comparing OS and PFS in cases with or without hepcidin gene alterations.

Supplementary Figure 4 GO analysis for hepcidin. **(A)** A heat map showing the top 50 genes negatively correlated with hepcidin in LUAD. **(B)** A heat map showing the top 50 genes negatively correlated with hepcidin in LUSC. **(C)** Top 20 enrichment terms in MF categories in LUAD. **(D)** Top 20 enrichment terms in MF categories in LUSC. **(E)** Top 20 enrichment terms in CC categories in LUAD. **(F)** Top 20 enrichment terms in CC categories in LUSC.

Supplementary Figure 5 Enrichment plots from GSEA. **(A, B)** A merged plot showing the pathways associated with hepcidin expression in LUAD and LUSC based on GO analyses. **(C, D)** A merged plot showing the pathways associated with hepcidin expression in LUAD and LUSC based on KEGG analyses.

Supplementary Figure 6 Scatterplots of correlations between hepcidin expression and infiltration levels of immune cells in LUAD.

Supplementary Figure 7 Scatterplots of correlations between hepcidin expression and infiltration levels of immune cells in LUSC.
