# Supplementary figures and images for: Hepcidin Upregulation in Lung Cancer: A Potential Therapeutic Target Associated With Immune Infiltration
Source: Front Immunol. 2021 Apr 1;12:612144. doi: 10.3389/fimmu.2021.612144 (PMC8047218; doi:10.3389/fimmu.2021.612144)

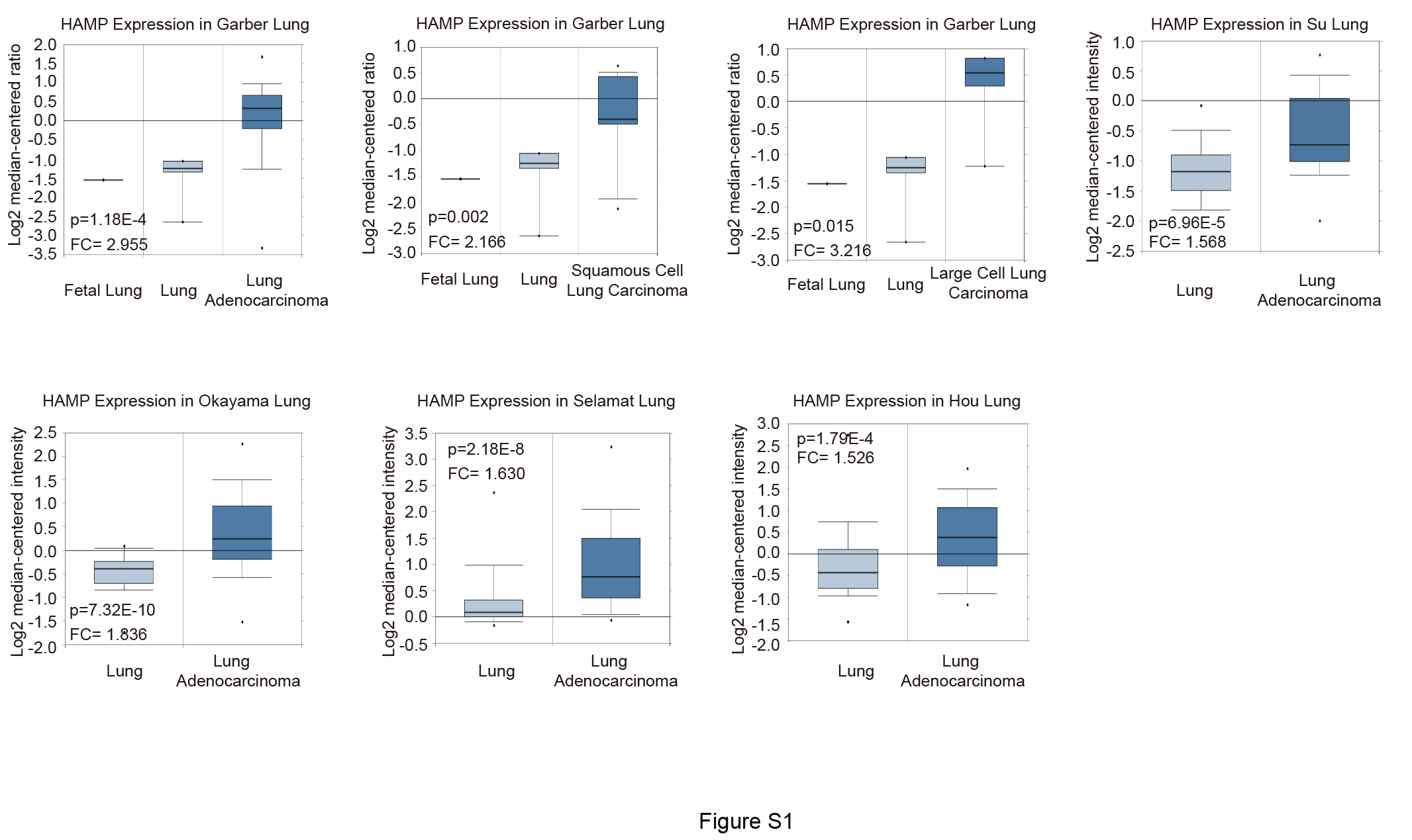

Supplement: Supplementary Figure 1 — Box plots comparing hepcidin expression in normal individuals and lung cancer patients obtained from the Oncomine database. [file Image_1.jpeg]

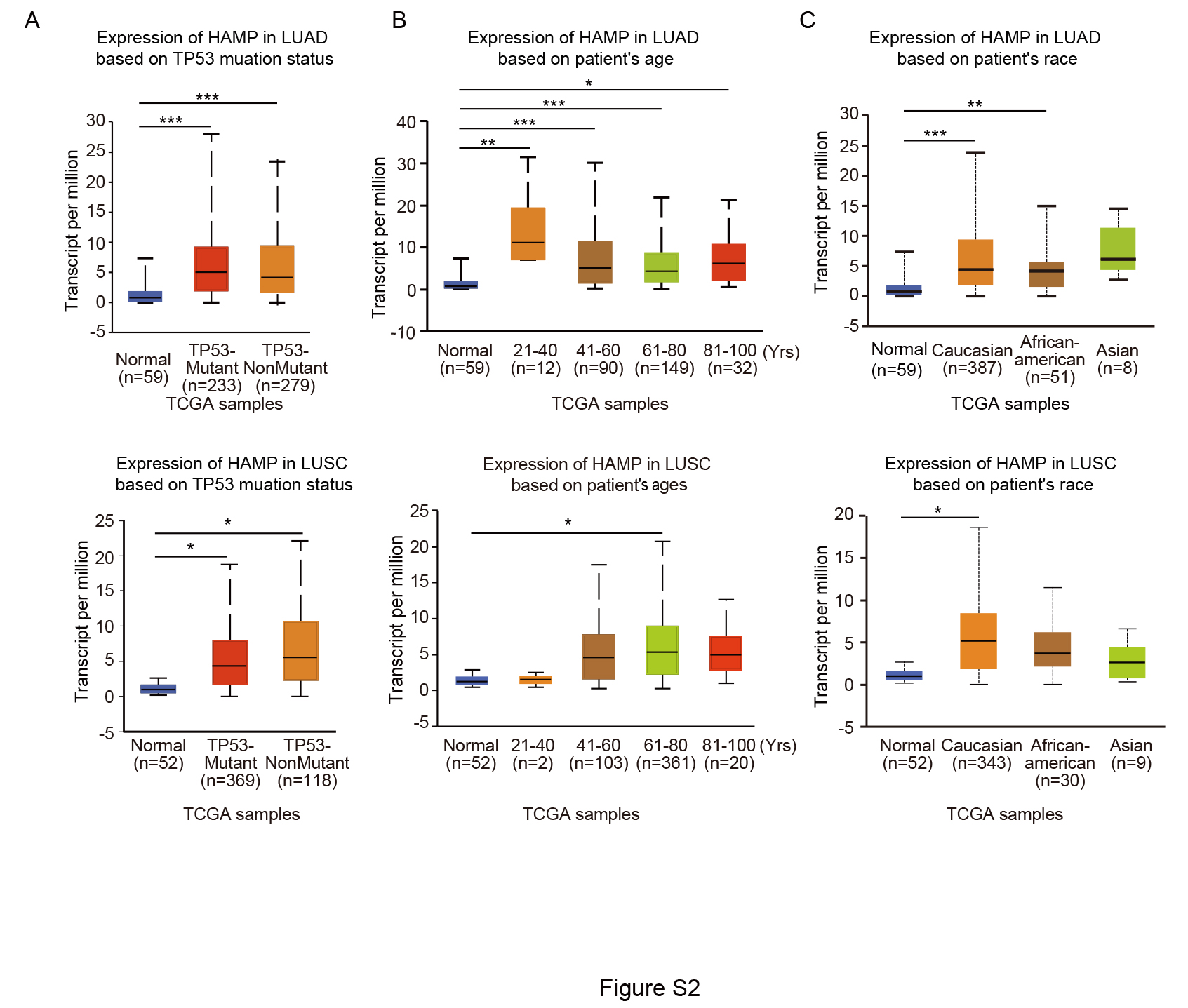

Supplement: Supplementary Figure 2 — Box plots evaluating hepcidin expression among different groups of patients based on clinical parameters using the UALCAN database. Analysis is shown for TP53 mutation status (A), age (B), and race (C). *p < 0.5, **p < 0.01, ***p < 0.001. [file Image_2.jpeg]

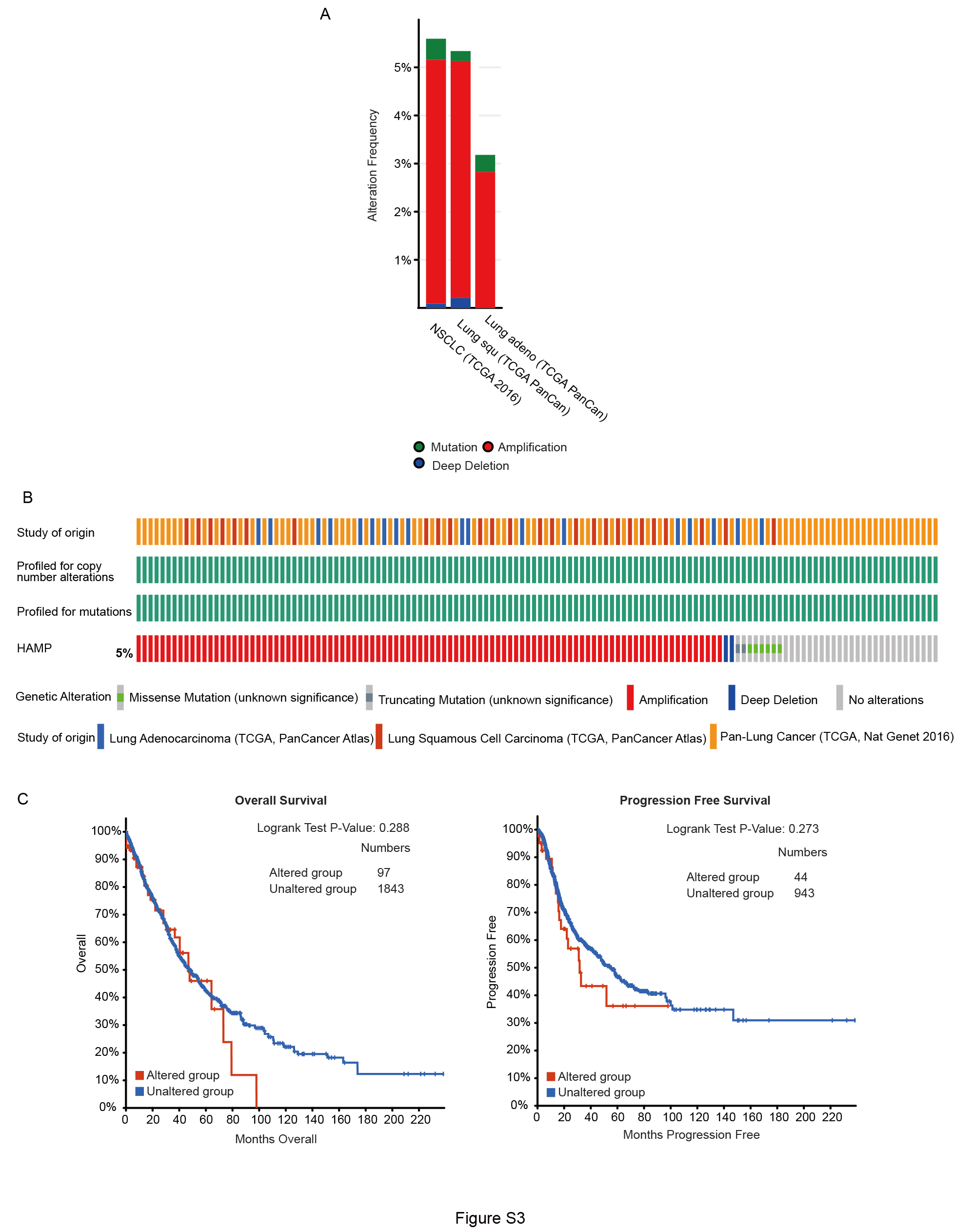

Supplement: Supplementary Figure 3 — Alteration frequency of hepcidin. (A) Summary of alterations in hepcidin. (B) OncoPrint visual summary of alterations in a query of hepcidin from cBioPortal. (C) Kaplan-Meier plots comparing OS and PFS in cases with or without hepcidin gene alterations. [file Image_3.jpeg]

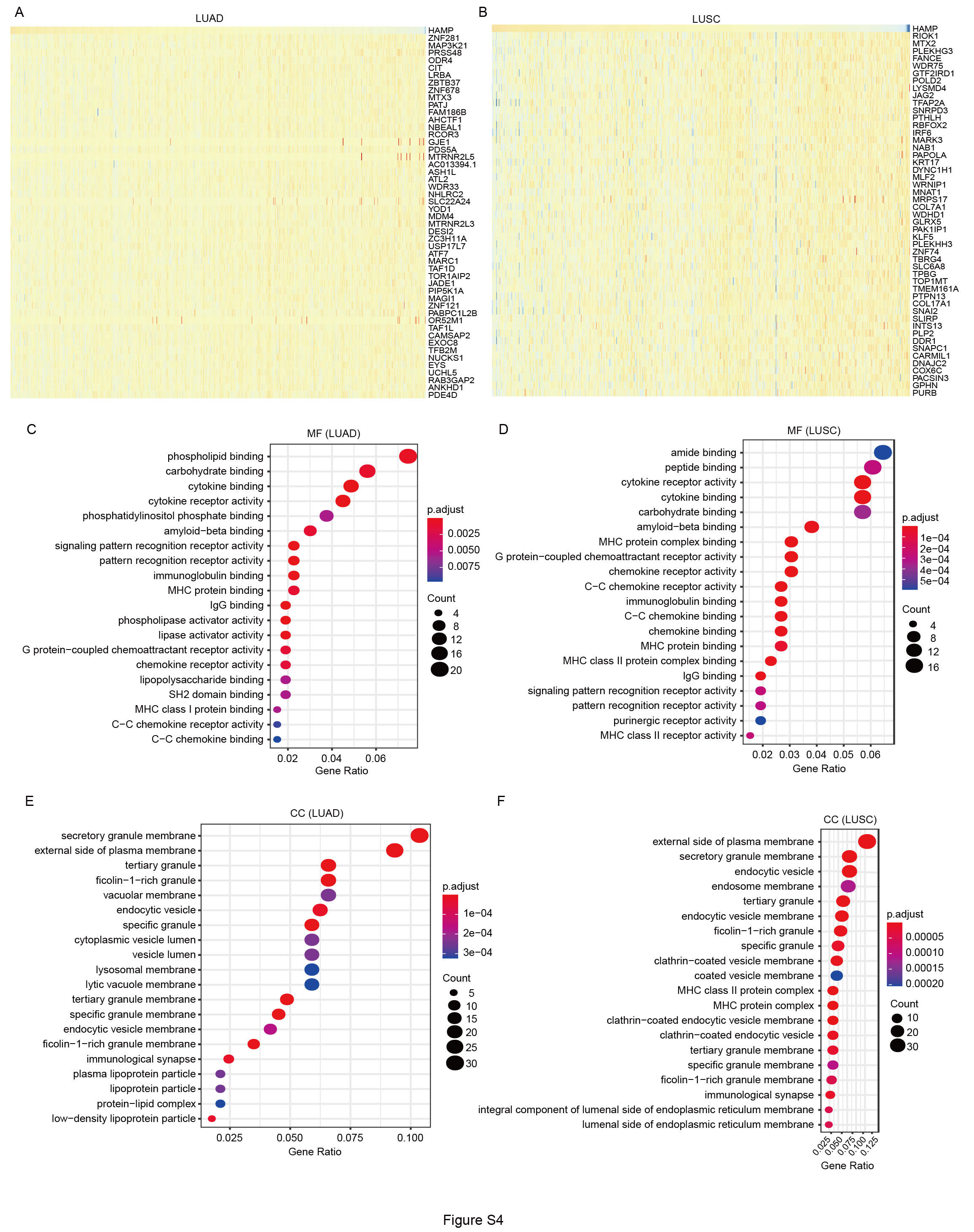

Supplement: Supplementary Figure 4 — GO analysis for hepcidin. (A) A heat map showing the top 50 genes negatively correlated with hepcidin in LUAD. (B) A heat map showing the top 50 genes negatively correlated with hepcidin in LUSC. (C) Top 20 enrichment terms in MF categories in LUAD. (D) Top 20 enrichment terms in MF categories in LUSC. (E) Top 20 enrichment terms in CC categories in LUAD. (F) Top 20 enrichment terms in CC categories in LUSC. [file Image_4.jpeg]

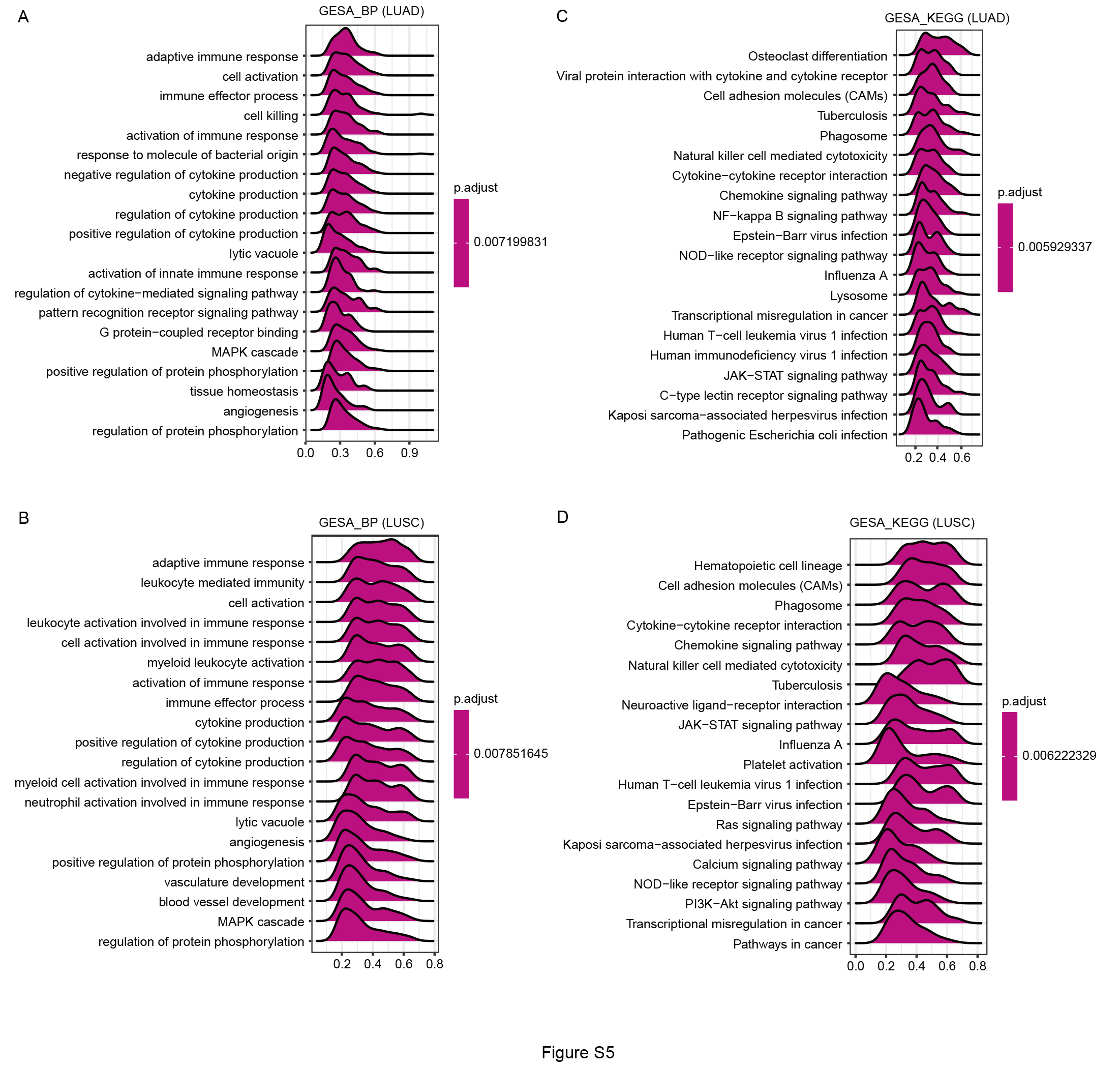

Supplement: Supplementary Figure 5 — Enrichment plots from GSEA. (A, B) A merged plot showing the pathways associated with hepcidin expression in LUAD and LUSC based on GO analyses. (C, D) A merged plot showing the pathways associated with hepcidin expression in LUAD and LUSC based on KEGG analyses. [file Image_5.jpeg]

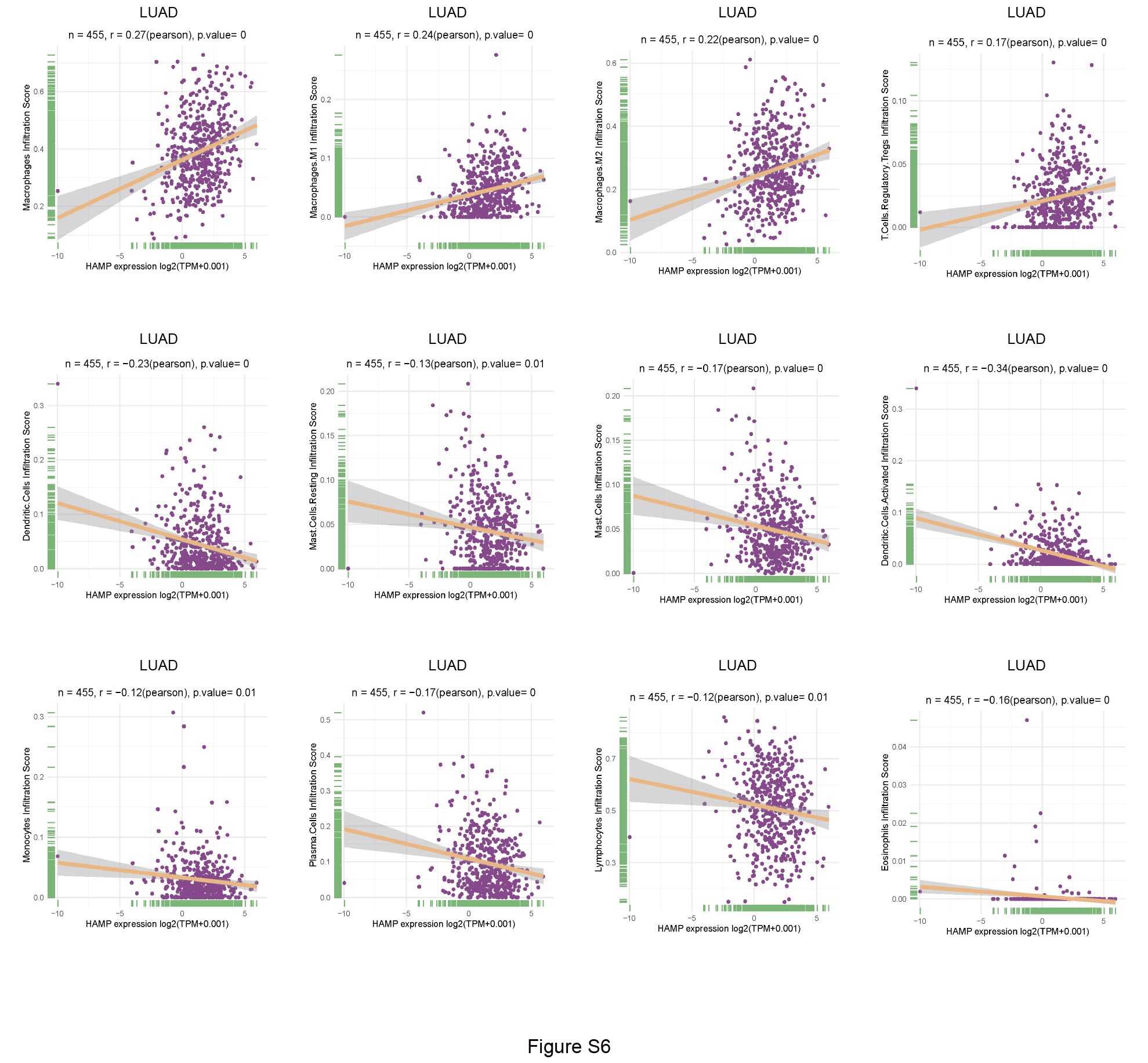

Supplement: Supplementary Figure 6 — Scatterplots of correlations between hepcidin expression and infiltration levels of immune cells in LUAD. [file Image_6.jpeg]

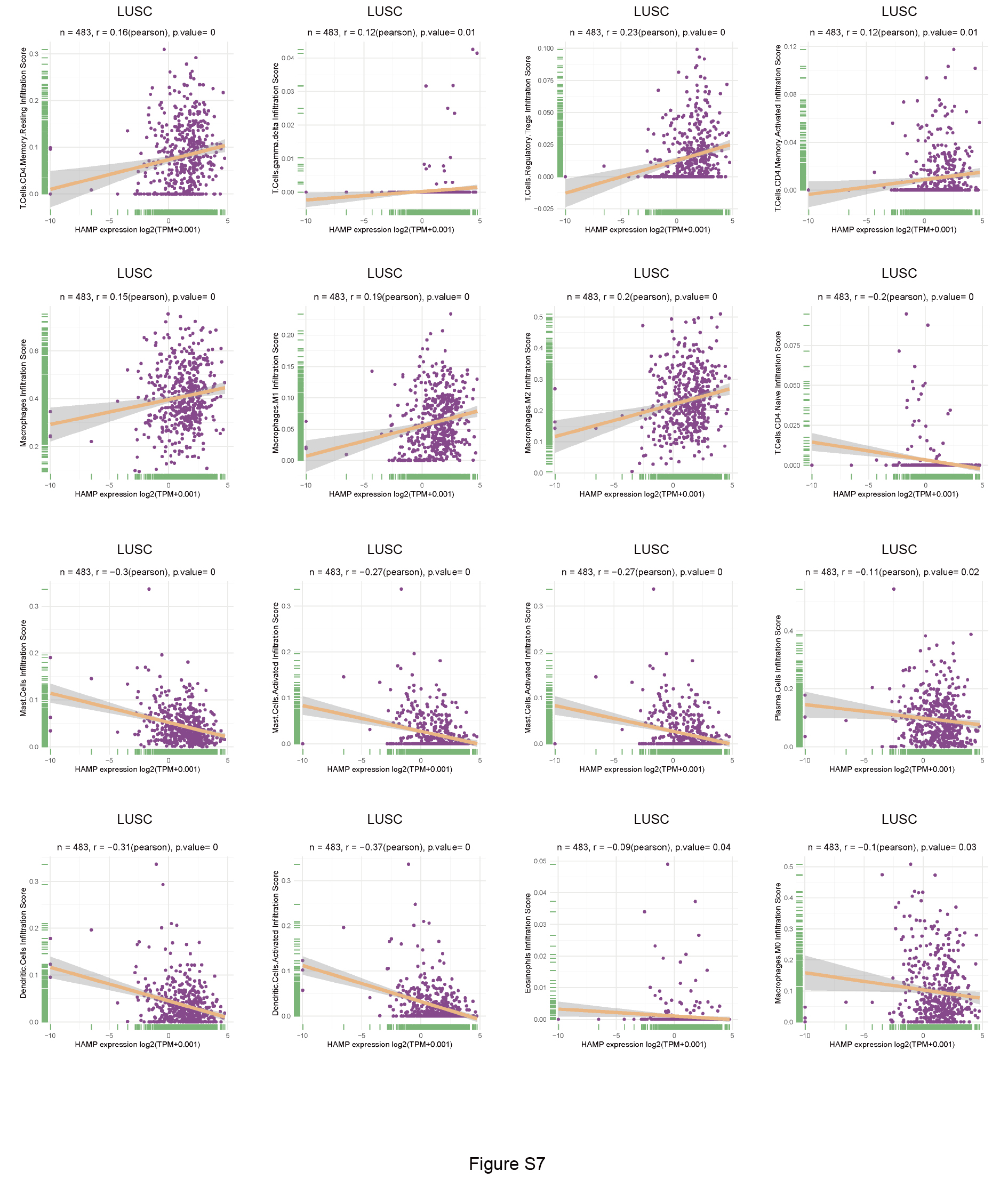

Supplement: Supplementary Figure 7 — Scatterplots of correlations between hepcidin expression and infiltration levels of immune cells in LUSC. [file Image_7.jpeg]
